# Diabetes in a hospital cohort of persons living with HIV: a descriptive and comparative study in French Guiana

**DOI:** 10.1186/s12879-023-08455-x

**Published:** 2023-07-13

**Authors:** Mathieu Nacher, Sebastien Rabier, Aude Lucarelli, Louise Hureau, Antoine Adenis, Nezha Hafsi, Nadia Sabbah

**Affiliations:** 1 CIC INSERM 1424, Centre Hospitalier de Cayenne, Cayenne, 97300 French Guiana; 2 DFR Santé, Université de Guyane, Cayenne, 97300 French Guiana; 3 COREVIH Guyane, Centre Hospitalier de Cayenne, Cayenne, 97300 French Guiana; 4 Service d’endocrinologie diabétologie, Centre Hospitalier de Cayenne, Cayenne, 97300 French Guiana

**Keywords:** HIV, Diabetes, Macrovascular complications, Microvascular complications, Opportunistic infections, Mortality

## Abstract

**Background:**

In French Guiana (population 294,000) the prevalence of type 2 diabetes (10%) and of HIV(1.1%) are very high. Our objective was to determine the prevalence of diabetes and its complications in a HIV cohort.

**Materials and methods:**

We enrolled HIV-infected persons followed in Cayenne, Kourou, and Saint Laurent du Maroni hospitals between January 1, 1992 and December 31, 2021 in the French Hospital Database for HIV (FHDH) a national database compiling data from all French regions.

**Results:**

There was no difference of diabetes prevalence between men (8.2%) and women (8.8%), P = 0.4. Patients with diabetes were older (56 years ± 13.4) than those without diabetes (44.7 years ± 13.6) and prevalence increased with age. The proportion of persons with diabetes was greater among virologically suppressed persons (10%) than those with a detectable viral load under antiretroviral treatment (5.8%). Persons with diabetes had substantially greater CD4 counts at diagnosis than persons without diabetes. The majority of macro and microvascular complications were observed in people with diabetes. Persons with diabetes and HIV were significantly less likely to have had AIDS (1.6 versus 2.2 per 100 person-years, respectively). Overall, 374 persons living with HIV of 4167 had died (9%) the proportion of persons with diabetes among the dead was greater than those who did not die 11.7% versus 8.1%, respectively, p = 0.017. However, persons with diabetes were older and hence died older, 62.3 years (SD = 1.9) for deceased persons with diabetes versus 50.4 years (SD = 0.8), P < 0.0001. However, using Cox regression to adjust for age, initial CD4 count, country of birth there was no significant difference in the Hazard for death between persons with diabetes and persons without diabetes (aHR = 0.99, 95%CI = 0.65–1.5), P = 0.9.

**Conclusions:**

The prevalence of diabetes in our HIV cohort was high. Persons with diabetes had greater CD4 counts, earlier care, and greater virological suppression than persons without diabetes. There were no significant differences between persons with diabetes and without diabetes in terms of survival.

## Introduction

French Guiana is a French overseas territory located between Brazil and Suriname. Its GDP per capita is the highest in Latin America, which attracts numerous immigrants in search of better economic prospects. Thus, 29% of the population and nearly half of adults are socially vulnerable foreigners. Overall, nearly half of the population is considered to be poor [1].

Cardiovascular diseases (often due to hypertension and diabetes) and HIV are among the top causes of premature deaths (< 65 years). French Guiana is the French overseas territory where the prevalence of HIV is highest [2]. It is estimated that the number of persons living with HIV exceeds 3200 for a total population of 294,000 [3].The transmission of HIV is mostly heterosexual and 3/4 of persons living with HIV are foreigners [4]. Precariousness and sexual vulnerability explain why a large proportion of infected immigrants acquire the virus after their arrival in French Guiana [5, 6]. Antiretrovirals and tests are free regardless of origin or socio-economic level. Undocumented immigrants with HIV have the right for residence permits and health insurance coverage. French Guiana is often viewed as a place where HIV and tropical diseases lead the burden of disease. However, the epidemiologic transition towards chronic diseases is largely completed [7, 8].Hence, the prevalence of diabetes in 2014 was estimated at 10% among the adult population of French Guiana and exceeded 20% among persons aged 45 years and above [9, 10]. We have shown that in French Guiana health inequalities are found for chronic diseases, infectious diseases, obstetrical problems; usually socially precarious persons present late, with more advanced diseases, and a greater risk of complications [11–14]. However, for diabetes or for HIV, once the persons are diagnosed and given care, the universal health care system tends to erase differences in outcome between precarious and non-precarious persons (defined by an EPICES score > 30) [15].Hence micro and macrovascular complications are not significantly different between precarious and non-precarious persons. Similarly, for HIV, although foreigners often get diagnosed later, once treated, there is no significant difference in AIDS incidence or survival.

Given the high prevalence of HIV and diabetes in French Guiana, the coexistence of both pathologies is relatively frequent. Furthermore, with the drastic decline in mortality, the HIV cohort ages and becomes increasingly vulnerable to cardiovascular diseases. Both diabetes and HIV infection are risk factors for infectious diseases. HIV treatments, notably antiprotease drugs, may trigger type 2 diabetes [16].Given the multiethnic nature of our HIV cohort, the ethnic specificities of diabetes, and the socioeconomic context, the aim of the study was to describe prevalence and incidence, clinical characteristics, and risk factors of diabetes mellitus among persons living with HIV living in French Guiana.

## Methods

### Study type

We conducted a retrospective cohort study on the HIV hospital cohort of French Guiana.

### The French Guiana Hospital database on HIV

After giving informed consent, adult HIV-infected persons with at least one consultation followed in Cayenne, Kourou, and Saint Laurent du Maroni hospitals between January 1, 1992 and December 31, 2021 were enrolled in the French Hospital Database for HIV (FHDH) a national database compiling data from all French regions.( [17, 18] This covers most adult in- and out-patients followed in French Guiana and nearly all AIDS cases. Recent estimates suggested that 85% of persons living with HIV were followed at the hospital centers [19]. Trained clinical research technicians entered demographic data, diagnoses using the 10th international classification of diseases, treatments, medical events, CD4 and CD8 counts, and HIV viral loads [18]. This data collection is focused on HIV not diabetes therefore the information on diabetes were scant. The specific variables collected were the following: age, sex, country of origin, date of HIV diagnosis, date of death, date of AIDS, date of each opportunistic infection, CD4 nadir, HIV viral load, antiretroviral treatment, date of diabetes, presence of a diagnosis that is a complication of diabetes (amputation, stroke, blindness, myocardial infarction, nephropathy, neuropathy).

### Statistical analysis

The main outcomes were prevalences of different infectious, micro and macrovascular complications, and incidence of AIDS, diabetes, and survival. First, we performed a descriptive analysis of the cohort. Qualitative variables were expressed as frequencies and percentages. Quantitative data were expressed as means and standard deviations or medians and interquartile ranges, as appropriate. We cross-tabulated the variables with presence/absence of diabetes and computed a Chi square test. Comparisons of quantitative variables between precarious and non-precarious persons were made using Student’s t-tests or Rank sum tests, as appropriate. We used regression analyses to model intervals between HIV diagnosis, specialized care, and antiretroviral treatment initiation while controlling for the period when diagnosis was given in 5 year-increments (recommendations varied in time). Survival analysis allowed to plot Kaplan Meier curves and Cox models were used to control for potential confounders. We used different failure criteria: death, AIDS, and diabetes. The statistical analysis was performed using STATA software ®(STATA®, College Station, Texas, USA). The significance level was 5%.

### Ethical and regulatory aspects

All persons living with HIV included in the FHDH give informed consent for the use of their anonymized data and for the publication of anonymized results. This cohort has been approved by the Commission Nationale Informatique et Libertés (27th of November CNIL 1991, subsequently updated March 30th 2021.) and published in the Journal Officiel (the official bulletin enacting all laws and decrees in France) on January 17th 1992 (JORF n° 0014 17/01/1992 ), and has led to over 400 international publications. By decree (JORF 0102, Décret n° 2017 − 682 du 28 avril 2017), in all French regions the COREVIH are the structures mandated to collect and analyze data and transmit yearly epidemiological reports to the ministry of health.

## Results

### Overall HIV cohort profile

The median age was 44 years (IQR = 36–55) and 48% were men. Among the cohort 32.2% of persons were Haitian, 21% were French, 19.1% were Surinamese, 9.7% Brazilian, 8% Guyanese, 2.1% Dominican, 3.7% other nationalities, and 4.2% of unknown nationality. Chronic hepatitis B prevalence among persons living with HIV was 4.4%, hepatitis C prevalence was 1.6%, and HTLV-1 prevalence was 4.7%.

### Persons with diabetes in the HIV cohort

**Fig. 1:**
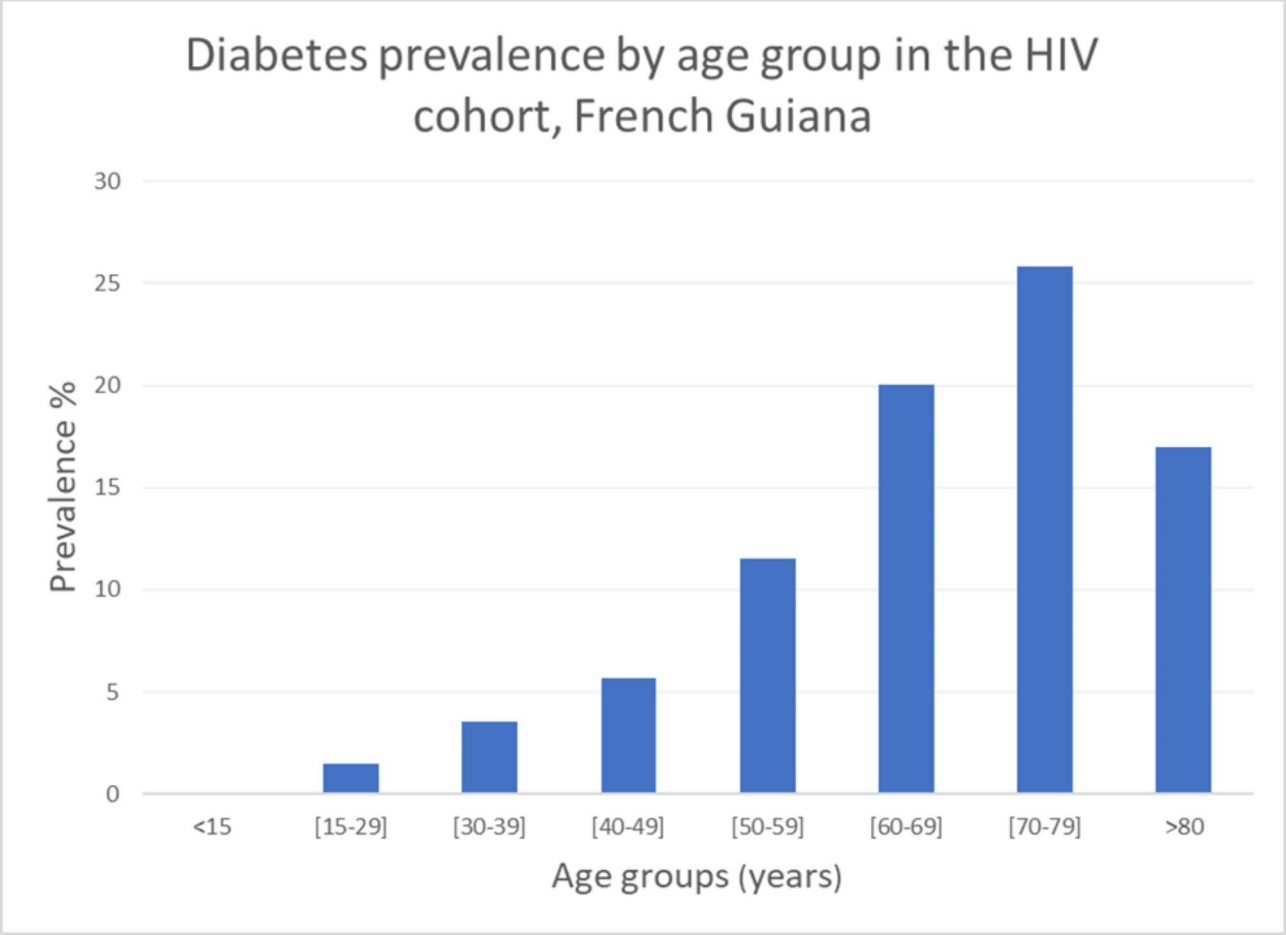
Diabetes prevalence -by age group in the VIH cohort, French Guiana

Overall, since the beginning of the HIV cohort, there were 353/4167 (8.5%) HIV-infected persons with diabetes. There was a preponderance of women in the HIV cohort: 2153 women vs. 1999 men, and 14 transgenders. There was no difference of diabetes prevalence between men (8.2%) and women (8.8%), P = 0.4. Persons with diabetes were older (56 years ± 13.4) than those without diabetes (44.7 years ± 13.6) and prevalence increased with age (Fig. 1). For those aged 45 or more, the prevalence was 12.8% in men and 14.3% in women, P = 0.33.

**Fig. 2:**
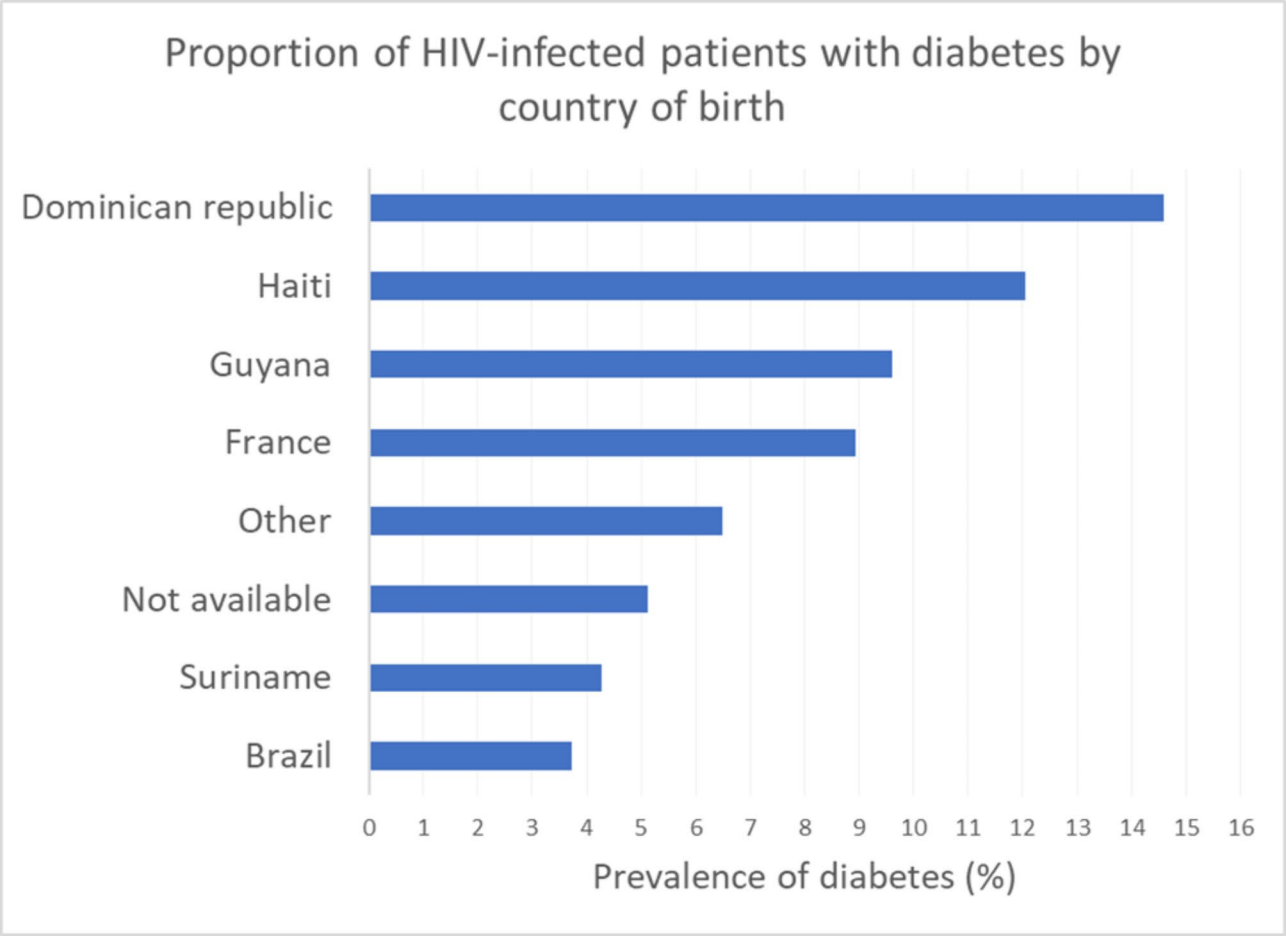
Proportion of persons living with HIV with diabetes by country of birth

Figure 2 shows that the prevalence of diabetes was greatest for persons from Haiti and the Dominican Republic, whereas it was lowest for those from Suriname and Brazil (P < 0.001).

### Virological suppression and diabetes

At the time of study, in early 2022 (N = 1971), the proportion of persons with diabetes was greater among virologically suppressed persons (10%) than those with a detectable viral load under antiretroviral treatment (5.8%) the crude odds ratio for therapeutic success and diabetes was 0.5 (95%CI = 0.31–0.95); P = 0.02.

### Opportunistic infections

Table 1 shows that persons with diabetes and HIV were significantly more likely to have had Esophageal candidiasis, pneumocystosis, and cerebral toxoplasmosis.

**Table 1:** Opportunist infections for people with or without diabetes

Infection	No diabetes (N = 3814)	Diabetes (N = 353)	Proportion of diabetes	P-value*
**Esophageal candidiasis**				0.0007
No	3568	317	8.16	
Yes	246	36	12.77	
**Cytomegalovirus**				0.346
No	3707	340	8.40	
Yes	107	13	10.83	
**Cryptococcal meningitis**				0.964
No	3754	346	8.44	
Yes	60	7	10.45	
**Histoplasmosis**				0.365
No	3479	327	8.59	
Yes	335	26	7.20	
**Pneumocystosis**				0.0003
No	3669	328	8.21	
Yes	145	25	14.71	
**Toxoplasmosis**				0.045
No	3609	325	8.26	
Yes	205	28	12.02	
**Tuberculosis**				0.429
No	3463	316	8.36	
Yes	351	37	9.54	

*Chi^2^ test

### Diabetes complications

Table 2 shows diabetes complications. We noticed a significant increase in the prevalence of macro and microvascular complications in the group with diabetes.

**Table 2:** Comparison of macro and microvascular complications between persons living with HIV with and without diabetes

	No diabetes (N = 3814)	Diabetes (N = 353)	Proportion of diabetes	P*
**Amputation**				0.001
No	3803	348	0.08	
Yes	11	5	0.31	
**Stroke**				0.001
No	3791	345	0.08	
Yes	23	8	0.26	
**Blindness**				0.101
No	3796	349	0.08	
Yes	18	4	0.18	
**Myocardial infarction**				< 0.001
No	3765	339	0.08	
Yes	49	14	0.22	
				< 0.001
**Nephropathy**	3768	340	0.08	
No	46	13	0.22	
Yes				
**Neuropathy**				< 0.001
No	3782	340	0.08	
Yes	32	13	0.29	

*Chi^2^ test.

### Intervals between HIV diagnosis care and antiretroviral treatment

**Fig. 3:**
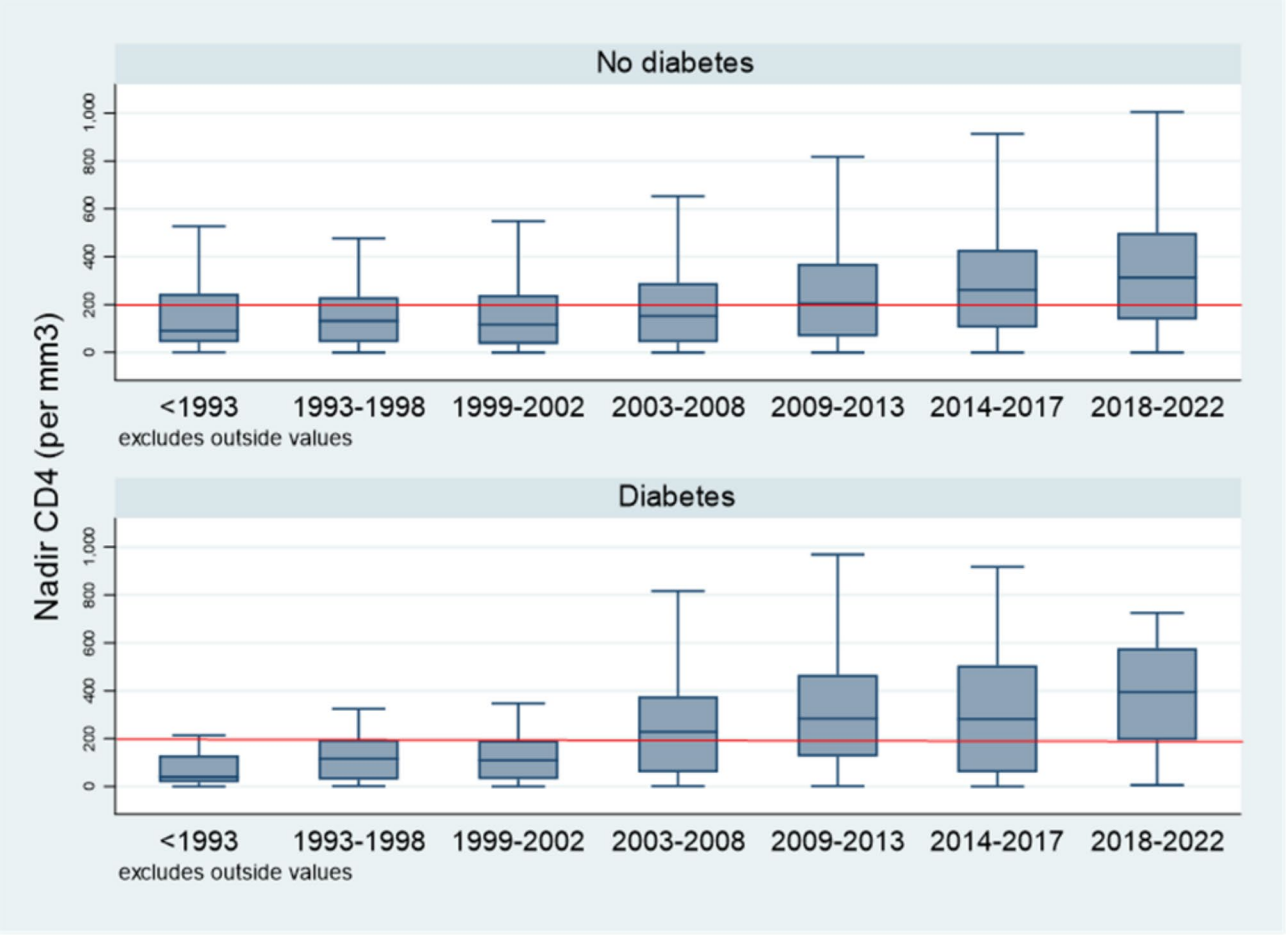
Nadir CD4 (per mm^3^) in patients with and without diabetes

After taking into account the period when the patient was diagnosed, the delay between HIV diagnosis and first consultation with an HIV specialist (not necessarily antiretroviral treatment) was significantly shorter among persons with diabetes − 0.46 years relative to persons without diabetes (P = 0.01). There was not significant difference between first specialized care and antiretroviral treatment initiation. The median CD4 count at the time of diagnosis was greater among persons with diabetes (381 cells/mm^3^, IQR = 164–577) than persons without diabetes (304 cells/mm^3^, IQR = 132–494), P = 0.009. The CD4 nadir was also greater among persons with diabetes relative to persons without diabetes after adjusting for period, the Nadir in persons with diabetes was 26 CD4 cells/mm^3^ higher than in persons without diabetes, P = 0.03 (Fig. 3).

### Incidence of AIDS

For AIDS, incidence rates were lower among persons with diabetes (incidence rate 1.6 per 100 person-years) than persons without diabetes (incidence rate 2.2 per 100 person-years), this was statistically significant both by using the Log Rank test (P = 0.0001) and using Cox regression to adjust for age, initial CD4 count, country of birth (aHR = 0.66, 95%CI = 0.45–0.97), P = 0.03.

For pneumocystosis, persons with diabetes had a slightly greater incidence rate (incidence rate 0.6 per 100 person-years) than persons without diabetes (incidence rate 0.4 per 100 person-years), Log Rank test (P = 0.04). However, after adjusting for CD4 counts, although the direction of the difference remained similar this was no longer significant (aHR = 0.8 95%CI = 0.6–1.1), P = 0.24. For cerebral toxoplasmosis, the significant relation between diabetes and toxoplasmosis was no longer significant in the crude and adjusted survival analyses.

For tuberculosis, there was no difference between persons living with HIV with diabetes and persons living with HIV without diabetes (incidence rate 0.9 per 100 person-years vs. 1.1 per 100 person-years), Log Rank test (P = 0.64).

### Survival and diabetes

Overall, 374 persons of 4167 had died (9%) the proportion of persons with diabetes among the dead was greater than those who did not die 11.7% versus 8.1%, respectively, p = 0.017. However, this is misleading because persons with diabetes were older and hence died older, 62.3 years (SD = 1.9) for deceased persons with diabetes versus 50.4 years (SD = 0.8), P < 0.0001. When looking at incidence rates, by contrast, persons with diabetes had a slightly greater survival (incidence rate 2.7 per 100 person-years) than persons without diabetes (incidence rate 3.7 per 100 person-years), but this was not statistically significant both by using the Log Rank test (P = 0.3) and using Cox regression to adjust for age, initial CD4 count, country of birth (aHR = 0.99, 95%CI = 0.65–1.5), P = 0.9.

### Incidence of diabetes

The incidence rate of diabetes among persons with HIV was 9.6 per 1000 person-years. Cox proportional hazards in a model with categorized age, weight, CD4 count nadir, and nationalities showed, unsurprisingly, that older age groups, heavier patients, and patients with CD4 counts at nadir > 500 cells/mm^3^ had a significantly greater incidence of diabetes than younger groups, lighter patients, and CD4 counts at nadir < 200 cells per mm^3^; persons from the Dominican republic had a greater incidence of diabetes adjusted hazard ratio = 3.4 (95%CI = 1.6-7), P = 0.001 relative to French nationals.

## Discussion

Here, in an HIV cohort including most patients since the beginning of the HIV epidemic in French Guiana, we show that the overall prevalence of diabetes was 8.5% and that persons with diabetes were on average over 11 years older than persons without diabetes. The prevalence seemed greater in women than in men 45 years or more, but this failed to reach statistical significance. In fact, in the general population of the territory, the prevalence of diabetes mellitus in women is 1.5 times higher than in men, but having HIV seemed to mitigate this difference [20]. This was slightly higher than what was observed in mainland France in the general population in 2015 (8.2% in women vs. 12.2% in men) [9]. In contrast to our results, several publications in HIV-infected populations from Africa show a higher prevalence of diabetes in men than in women [21, 22]. It was much higher than in a study in persons living with HIV in which the crude prevalence was 3.9% [23]. The mean age was substantially lower in French Guiana (56 years) than in mainland France (65 years). This presumably reflected differences in the ethnic make-up with persons of African ancestry who usually develop diabetes at a younger age than Caucasians [24, 25]. The prevalence of diabetes was greatest for persons from Haiti and the Dominican Republic, whereas it was lowest for those from Suriname and Brazil. As for age at diagnosis, there are known ethnic differences for the risk of diabetes, with persons of African ancestry being more at risk than Caucasians; however, although the explanations holds for Haitians and Dominicans, it does not for Surinamese who are mostly Maroons. Perhaps, for Brazilians and Surinamese, this corresponds to populations living along the border rivers where intestinal parasites are common, [26] and obesity is less common than in poor urban areas [27–29]. In Suriname, however, there are many variations in the metabolic syndrome and the prevalence of type 2 diabetes depending on ethnicity [30]. The prevalence of metabolic syndrome is highest for the Hindustanis (52.7%) and lowest for Maroons (24.2%) [31]. A study published in 2016 found an 11% prevalence of diabetes mellitus in the Dominican Republic in a population with HIV (6% had diabetes at the initiation of ART and 5% became diabetic after its introduction) [32]. The greater prevalence in the population of Dominican origin is perhaps due to the more sedentary lifestyle in French Guiana, but this impression would require formal testing. In this complex region, the singularities of diabetes in the general population and in the HIV population still need further exploration to disentangle the complex interactions between host genome, social and nutritional factors, and access to care.

Overall, a surprising fact was that when controlling for age and CD4 count the incidence of AIDS was lower among persons with diabetes than among persons without diabetes and that there was no difference regarding survival. Indeed, it would have been expected that persons with diabetes would cumulate the complications of HIV infections and those of diabetes. What seemed to happen was that, perhaps because they were followed for diabetes, they were seen earlier than persons without diabetes in terms of CD4 decline –nearly half a year on average and 80 CD4 cells/mm^3^ more—and this may have led to some gains regarding AIDS incidence and related deaths compensating the mortality due to diabetes. Since in most cases, the cause of death was not known, we can only speculate to explain this. Interestingly, persons with diabetes seemed more likely to have Esophageal candidiasis and pneumocystosis than persons without diabetes. A study published in 2015 found HIV infection and corticosteroid use, especially at higher doses, were independently associated with Esophageal candidiasis, but not diabetes[33], the Ogiso study shows that diabetes is an important risk factor for esophageal candidiasis. [34] Our study found an increase in esophagitis when HIV is associated with diabetes. This was not a very large nor significant effect but it seems plausible. Naturally, among persons with diabetes, a history of cardiovascular complications was substantially more frequent than among persons without diabetes.

Persons with diabetes were more likely to be virologically suppressed than persons without diabetes. Although this could partly reflect the role of antiretrovirals in triggering diabetes, we suspect that this reflects the greater adherence and follow up of diabetic persons because they often require more complex care for 2 chronic diseases.

The present study has a number of limitations. The database is dedicated to HIV care, thus it lacked important aspects of the features of the person’s diabetes. Body mass index was not available, the exact date of diabetes discovery was not available, some of the significant complications of diabetes, metabolic aspects, and cause of death were not available. Some patients may have died unbeknownst to us after right censoring and thus we may be missing some information in a territory where migration is intense. We did not study the timing and type of antiretroviral drugs used which are known to be associated with diabetes, but our aim was more descriptive and broad scale.

The long time span covered involves biases due to changing practices, gradually improving treatments and diagnostic capacity that are difficult to control for, even if we tried to do so. Nevertheless, this large study has the merit of covering the majority of persons living with HIV ever followed and gives information that may be of use for both HIV and diabetes specialists at a time when cohorts age, when most persons living with HIV are virologically suppressed and chronic diseases become more important than opportunistic infections.

In conclusion, in this historical cohort of persons living with HIV, although persons with diabetes had a greater proportion of macro and microangiopathic complications, there were no significant differences between persons with and without diabetes in terms of survival, and persons with diabetes had a lower incidence of AIDS. Despite this counterintuitive finding, esophageal candidiasis and pneumocystosis seemed to be more frequent among persons with diabetes than persons without diabetes. The prevalence of diabetes was high but slightly lower than previous reports in French Guiana and it differed between different nationalities.

## Data Availability

The datasets generated during and analyzed during the current study are not publicly available due to the fact that data transfer from one center to another requires permission from the Commission Nationale Informatique et Libertés (CNIL) but are available from the corresponding author on reasonable request after approval from the Commission Nationale Informatique et Libertés (CNIL).

## Declarations

### Methods

Methods were performed in accordance with French national guidelines and regulations.
